# Adapting the Quebecois method for assessing implementation to the French National Alzheimer Plan 2008–2012: lessons for gerontological services integration

**DOI:** 10.5334/ijic.1201

**Published:** 2014-06-16

**Authors:** Dominique Somme, Hélène Trouvé, Catherine Perisset, Aline Corvol, Joël Ankri, Olivier Saint-Jean, Matthieu de Stampa

**Affiliations:** Department of Geriatrics, Université de Rennes 1, Rennes, France; Center for Research on Political Action in Europe (UMR 6051, CNRS), Université Rennes 1, Rennes, France; Fondation Nationale de Gérontologie, Paris, France; Caisse Nationale de Solidarité pour l'Autonomie, Paris, France; CHU de Rennes, Rennes, France; Department of Geriatrics, Université de Versailles–Saint-Quentin, Laboratoire Santé Environnement Vieillissement, EA 2506, Centre de Gérontologie, Paris, France; Department of Geriatrics, Université Paris-Descartes, Paris, France; Université de Versailles–Saint-Quentin, Laboratoire Santé Environnement Vieillissement, EA 2506, Centre de Gérontologie, Paris, France

**Keywords:** method of integrated care, assessment of implementation, geriatric services integration

## Abstract

**Introduction:**

Many countries face ageing-related demographic and epidemiological challenges, notably neurodegenerative disorders, due to the multiple care services they require, thereby pleading for a more integrated system of care. The integrated Quebecois method issued from the Programme of Research to Integrate Services for the Maintenance of Autonomy inspired a French pilot experiment and the National Alzheimer Plan 2008–2012. Programme of Research to Integrate Services for the Maintenance of Autonomy method implementation was rated with an evaluation grid adapted to assess its successive degrees of completion.

**Discussion:**

The approaching end of the president's term led to the method's institutionalization (2011–2012), before the implementation study ended. When the government changed, the study was interrupted. The results extracted from that ‘lost’ study (presented herein) have, nonetheless, ‘found’ some key lessons.

**Key lessons/conclusion:**

It was possible to implement a Quebecois integrated-care method in France. We describe the lessons and pitfalls encountered in adapting this evaluation tool. This process is necessarily multidisciplinary and requires a test phase. A simple tool for quantitative assessment of integration was obtained. The first assessment of the tool was unsatisfactory but requires further studies. In the meantime, we recommend using mixed methodologies to assess the services integration level.

## Introduction

Services integration is often cited as a way to adapt heath care systems to the changing demographic and epidemiological stakes [1, 2]. However, the methods enabling implementation of new organizations are rarely spelled-out clearly. Moreover, the need to follow integration-implementation progression is considered one of the major challenges in this research field [3]. The Quebecois Programme of Research to Integrate Services for the Maintenance of Autonomy method experiments, followed by their becoming public policy there and then in France, provided the opportunity to evaluate the degree of implementation. Herein, we describe the lessons learned concerning assessment of the degree of implementation, from the comparison of the Programme of Research to Integrate Services for the Maintenance of Autonomy method quantitative and qualitative data. That study was prematurely interrupted when the institutionalization phase began because of political pressure as the president's term ended, hence the results presented herein must be confirmed in other contexts.

## Context: experiments based on the Programme of Research to Integrate Services for the Maintenance of Autonomy integration method

### Programme of Research to Integrate Services for the Maintenance of Autonomy method: definition and origin

The Programme of Research to Integrate Services for the Maintenance of Autonomy method is based on this definition of integration. ‘We consider integrated care to be a discrete set of techniques and organizational methods designed to create connectivity, alignment and collaboration within and between the cure and care sectors at the funding, administrative and/or provider levels. The goals are to enhance quality of care and quality of life, consumer satisfaction, and system efficiency [4]’. More precisely, this method seeks to instigate system change via the approach Leutz designated ‘coordination’ [5, 6]. Starting with the study of these definitions and concepts, and the systematic analysis of the literature, the Programme of Research to Integrate Services for the Maintenance of Autonomy method is based on six elements: inter-organization and inter-facility coordination, a single entry point, case-management system, a sole evaluation tool coupled to a classification-of-needs system, use of an individualized-services plan and an information-sharing system with a shared electronic clinical chart [5]. Modelized in Quebec [5], tested between 2000 and 2005, and evaluated positively [7], Programme of Research to Integrate Services for the Maintenance of Autonomy inspired the 2004 reform of that national health care system [7].

### First application of the method outside its country of origin: Programme of Research to Integrate Services for the Maintenance of Autonomy-France

#### Experimental phase

##### The Programme of Research to Integrate Services for the Maintenance of Autonomy-France experiment

Based on the promising Quebecois Programme of Research to Integrate Services for the Maintenance of Autonomy experimentation results, French authorities funded the Programme of Research to Integrate Services for the Maintenance of Autonomy-France project (2006–2010), aiming to reproduce the method in France and testing its appropriateness for this different context [8, 9]. Three geographic areas differing in their populations and accesses to care were selected for this experiment. All stakeholders involved in keeping the elderly in their own homes were invited to participate in this integration experiment directed by a Steering Committee in close contact with the scientific team. The organizational modifications required to initiate the Programme of Research to Integrate Services for the Maintenance of Autonomy method were identified, and the Steering Committee followed and facilitated their operationalization. The experiment's midterm outcome was promising but still required a major integrative effort [10–12]. Measuring impact was not foreseen. However, the programme assured that the services organizations created were truly innovative and changed professional practices [13], and that this experiment could make sense for the most vulnerable users [12].

##### Home for Autonomy and Integration of Alzheimer patients experiments

Based on the first Programme of Research to Integrate Services for the Maintenance of Autonomy-France experiment, public authorities decided, in 2008, to repeat the experiment on a larger scale, within the framework of the National Alzheimer Plan 2008–2012, and called it Home for Autonomy and Integration of Alzheimer patients (MAIA) [8]. Among the plans’ 44 objectives, 10 were considered core aims, one of which was to develop integrated care, based on the Programme of Research to Integrate Services for the Maintenance of Autonomy method renamed Home for Autonomy and Integration of Alzheimer patients [14].

Unfortunately, that name is doubly misleading as no facility construction (‘homes’) was involved; only the six Programme of Research to Integrate Services for the Maintenance of Autonomy method axes were established. Moreover, the method was not reserved for Alzheimer's patients, merely considered a ‘typical case’ of system fragmentation, but that the Programme of Research to Integrate Services for the Maintenance of Autonomy method should be tried for Alzheimer's patients and subsequently be ‘expanded’ to all people with loss of autonomy. The experiment covered 17 geographic areas with very diverse sizes and population densities, and was conducted jointly by a national Steering Committee and a local director in each zone called the ‘local pilot’ [8]. The pilot in one area did not provide the requested indicators. However, the remaining 16 selected zones were home to 4,720,775 inhabitants, among which 982,779 were aged 60 years and over and 65,051 were eligible for financial assistance to pay for services to compensate for the loss of functional autonomy. In those areas, based on an extrapolation of prevalence calculated from reimbursement claims made to the National Health Insurance, an estimated 79,211–103,221 people had Alzheimer's disease. The integrated method's target population was all persons with loss of autonomy, particularly those with Alzheimer's disease. Additional ‘characteristics’ were not required for inclusion in that very large target population. However, case management was intended to benefit only a very small subpopulation included in the programme whose ‘home-care situation’ was complex, but the latter was not explicitly defined, and relied rather on the judgments of implicated professionals and case managers. Hence, approximately 40 individuals were selected for case management per manager (two case managers/site). All the health-and-social services (geriatric unit, geriatric day hospital, at-home hospitalization, long-term nursing services, professional and non-professional aides for personal autonomy, household services, etc.) required for community-dwelling elderly in the selected areas were concerned.

The 2008–2010 experiment was considered sufficiently satisfactory to undertake a progressive nationwide policy-extension process: adding 40 new geographic areas in 2011, 100 in 2012 and 50 in 2013.

## Innovations: methods for evaluating the degree of integration implementation

Here, we successively describe a grid developed in Quebec for research to measure integration and its conceptual framework, its adaptation to the French research setting and, finally, two tools (one long and one very short), with the latter developed for change-management purposes.

### The Quebecois Programme of Research to Integrate Services for the Maintenance of Autonomy experimental phase

A grid constructed to monitor method implementation relied on data from focus groups, composed of researchers, clinicians, facility administrators and government officials. The conceptual framework is the definition of integrated care (see above) and the six above-mentioned elements: inter-organization and inter-facility coordination, single entry point, case-management, a sole evaluation tool, use of an individualized-services plan and an information-sharing system [5]. This tool was developed to identify pertinent implementation-monitoring markers for each of the six system axes and establish their relative weighting, using precise indicators able to assess the implementation level for each of them. Variable weighting enables subsequent calculation of the final implementation rate. The implementation level, evaluated over 30 months by the research team, was reported to all the participants and discussed to consensus at roundtable meetings [15]. Stakeholders considered those reports to be a factor of the experiment's success [16]. The grid was complex and required the collection of quantitative (e.g. extraction of information from the case manager's files) and qualitative data (e.g. derived from participatory observation or the coordination-meeting report). This assessment method was doubly ‘validated’: as a function of its implementation stage, an integration-induced change could be determined (‘dose–response’ effect) and, for integration to produce its positive effects, i.e. preventing loss of autonomy (primary endpoint), the evaluation had to reach 70% implementation in Quebec (‘threshold’ effect) [7]. In 2005, based on those findings and inspired by the Programme of Research to Integrate Services for the Maintenance of Autonomy method, public authorities initiated an integration policy at the province level, through the Network of Integrated Services for the Elderly. During the latter's establishment, a simplified assessment tool (not shown here), using items considered in the Programme of Research to Integrate Services for the Maintenance of Autonomy method, was used. The Netword of Integrated Services for the Elderly-implantation monitoring tool was given to agencies for an obligatory annual self-evaluation [17].

### During the Programme of Research to Integrate Services for the Maintenance of Autonomy-France experimental phase

The Quebecois grid for assessing integration was adapted to the French context. The detailed grid modifications were published elsewhere [9, 17]. Each indicator was attributed a number of points, for a total of 100 points, yielding the implementation rate (Table 1). Close collaboration of French and Quebecois research teams made this adaptation possible. Concerning the method, we wanted to retain the component-weighting used during the Quebecois Programme of Research to Integrate Services for the Maintenance of Autonomy study and to revise, as needed, the indicators used.

As in Quebec, the research team assesses the rate with qualitative and quantitative data collected every 6 months, with results are reported annually to the stakeholders. The latter identified accompaniment by a research team as a success factor, even though none spontaneously cited rate assessment as a distinctive aspect of this support [12].

### The French National Alzheimer Plan 2008–2012: the Home for Autonomy and Integration of Alzheimer patients experimental phase

The evaluation grid to assess integration developed for the Programme of Research to Integrate Services for the Maintenance of Autonomy-France experiment underwent two changes during Home for Autonomy and Integration of Alzheimer patients. In both cases, the goal was to obtain a tool that did not require a research protocol to be completed.

One of the tools developed was aimed at accompanying the individuals responsible for carrying out the project (the pilots) in their work, enabling them to appreciate the degree of integration progression. This tool was derived directly from the evaluation grid and required massive data collection. It was given to the pilots during the institutionalization phase after 2010, but its real use had not been followed.

The other tool was developed to be a very simple way to evaluate integration based on the absence or presence of 24 criteria (Appendix A). Called the ‘MAIA 24’ score, its primary aim is to enable off-site external evaluation of the integration level, based on the key integration-progression indicators, eventually enabling comparisons among territories. Unlike the grid that seeks exhaustivity concerning all the key integration components, the ‘MAIA 24’ score focuses on several criteria that appear to be the most important and, thus, this simple tool has no pedagogical goal (for the pilot, stakeholders or deciders) concerning integration. Criteria were selected by interdisciplinary researchers and managers from health and social fields and by consensus. Consensus was reached easily through dialogue, without any particular method (e.g. nominal group or Delphi). The ‘MAIA 24’ criteria were selected from among the evaluation-grid items based on these characteristics: simplicity, restricted number, objective (as opposed to subjective) criteria, and those discriminating, as much as possible, criteria among the sites and their advances towards integration. Based on the pilots’ declaration in stage reports, the 17 experimental sites were monitored in parallel with this indicator and, more profoundly, by the national Steering Committee and two public health researchers. After refining the score during the second half of the experiment, it was possible to ‘classify’ the sites according to their evolution-towards-integration status and to adapt the score according to the deciders’ possible recommendations. Actual tool use by these deciders remains unknown because the scientific evaluation of implementation was interrupted.

## Results of the National Alzheimer Plan experimental phase and proposed score

**Figure 1.**
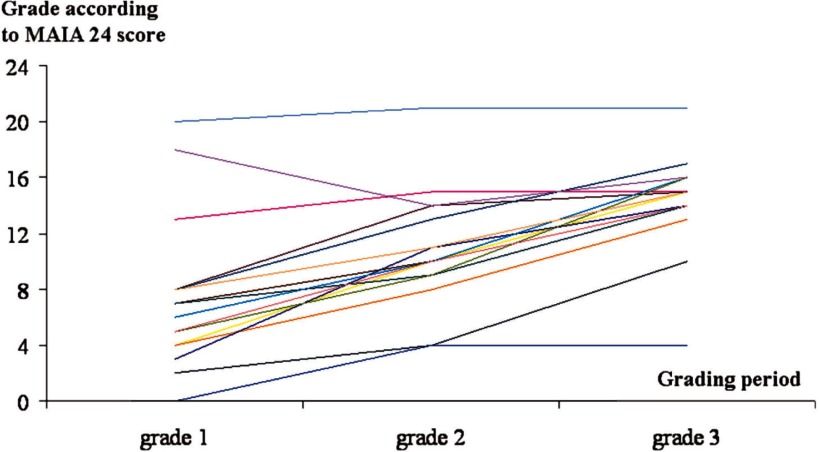
Successive ratings of the 17 sites using the described 24-point score. The ordinate is the grade accorded with this tool (maximum 24) plotted versus the grading period. Each line represents one site's three grades during the experimental period.

The rating was done with the ‘MAIA 24’ grid, with only objective data reported by the pilots to the national team at implementation onset (June 2009), during the preparatory phase of case management (October 2009) and at 1 year (June 2010). The evolving site profiles are illustrated in Figure 1. Three essential findings were drawn from this figure: globally, integration progressed at all the sites; rating dispersion tended to decline over time, which seems to indicate a trend towards homogeneity of the structures put in place (and, thus, contribute to the equitable treatment of the populations among the sites); and the score seems to have reached a marked ‘ceiling’ effect.

Using this grading method, five categories, 1–5, were identified, with 1 corresponding to the most advanced sites and 5 the least advanced. In addition to this grading, between April and July 2010, the national experts visited all the sites, where they conducted qualitative research with extensive on-site interviews with the pilot and his employer, observed the process put in place to orient ‘client’ requests for information as a single entry point and met all the case managers in focus groups. In addition, the experts discussed the on-site situation before the visits with the Steering Committee. After the on-site visits and taking into consideration all the available information, the two experts determined site typology, as a function of the status of their Home for Autonomy and Integration of Alzheimer patients-programme implementation advancement. Five new site groups, 1–5, were created, with 1 corresponding to the most advancement and 5 to the least.

Comparison of the grades of the two site-classification methods (Table 2) highlighted two principal findings: a relationship seemed to exist between the two assessment methods; and the relationship was not perfect, with some sites having high MAIA-24 score integration and low expert-accorded scores (objective data favouring integration but the real situation demonstrating poor partnerships), and some sites having the opposite ratings (objective data indicating low-level integration countered by the quantitative evaluation). The exact reasons for the discrepancies between the two rating methodologies were not completely elucidated. One possible explanation is that some pilots were more skilled in reporting and communication with regulatory authorities, while others had better personal approaches, empathy and local networking. A quantitative methodology would have favoured the former and a qualitative one the latter. Another possibility is the heterogeneity of integrated care among areas. In large experimental areas, the integrated-care level was constantly heterogeneous, with some territorial zones having effectively working integrated health- and social services, while fragmentation persisted in others. Depending on the indicator and the degree of heterogeneity, this disparity could engender over- or underestimation of the real integrated-care level. This hypothesis is supported by the link between area size and extent of the differences between the two assessment methods (data not shown).

Considering the results obtained during the first period, the final score at the end of the experimentation (2008–2011) led to a second evaluation (data not given but similar to those obtained during the first period, with a less marked ceiling effect; Appendix B).

## Why this study was not pursued

According to our findings, it is clear that the proposed simple tool must undergo serious validation and it is not yet possible to know whether or not the grid's larger pedagogical objective has been met. Experimental phase results were judged sufficiently convincing to initiate the institutionalization phase. However, the newly elected government's budget no longer funded research, which was interrupted.

The tools described herein were proposed on a larger scale in France, without any new study on validation or current practices planned. We explained elsewhere why an idea-to-outcome gap is common in politics because of the narrowness of the ‘political window’ during which any system change is possible [18]. Considering that integrated care is somewhat difficult to achieve and that ‘political discourse’ no longer supports the Home for Autonomy and Integration of Alzheimer patients approach, the new Home for Autonomy and Integration of Alzheimer patients tend to be reduced to only the implementation of a case-management initiative for the elderly in complex-living situations and some coordination mechanisms [18, 19].

## Conclusion: lessons drawn from the evaluation of the degree of implementation; relevance for the international community

### Positive lessons from the international Programme of Research to Integrate Services for the Maintenance of Autonomy method implementation experiment

The Programme of Research to Integrate Services for the Maintenance of Autonomy-France experiment and its institutionalization within the framework of the National Alzheimer Plan 2008–2012 constitute highly original sources of knowledge. Indeed, because the same integration implementation method was applied in France and Quebec, it is possible, for the first time, to obtain transnational data on integrations and to accumulate information on the assessment of the extent of integration implementation. All our results [8, 10–12] underline that the Programme of Research to Integrate Services for the Maintenance of Autonomy method is an adaptive methodology to move towards integrated care in systems as different as France (predominantly Bismarkian) and Quebec (predominantly Beveridgian) [14]. Our findings also stress the importance of political discourse to obtain the expected national impact of the policy. Finally, should another country decide to adopt and adapt the Programme of Research to Integrate Services for the Maintenance of Autonomy method to implement integrated care using the previously learned lessons [7, 11–13, 18], this paper, based on an interrupted implementation study, can help identify some additional key lessons.

### Positive and caution lessons from assessing implementation

Implementation-level evaluation:

1. constitutes an important condition supporting the organization modifications necessary to establish such an institutional, organizational and clinical innovation;
2. is possible with the Programme of Research to Integrate Services for the Maintenance of Autonomy methodology and enables implementation assessment in very different national settings, provided that the prerequisite adaptation work is done;
3. can be useful for two reasons: integration progression and, in this indication, its assessment can be simplified (but this approach must be repeated to be validated), and the evaluation may have an educational role, making it necessary to conserve a sufficiently subtle and, thus, rather extensive grid (this educational goal also warrants further work);
4. must not be the sole means of assessing real modifications engendered by integration implementation, even though it allows ‘regular’ off-site management, as it cannot replace more precise evaluation by on-site visits and meetings with the different partners involved; and
5. must be accompanied by adequate training of the individuals responsible for collecting and processing the data needed to provide essential information to services stakeholders, managers, administrators, policy makers and users.

## Figures and Tables

**Table 1.**
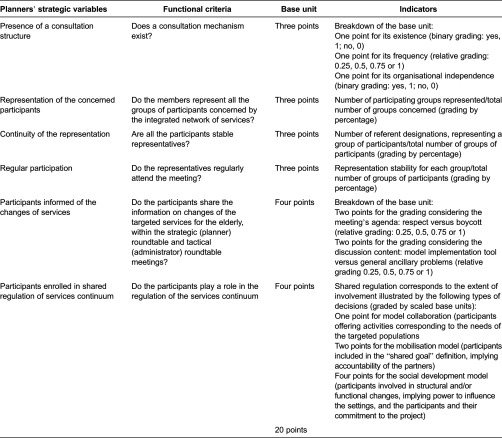
Example of the Programme of Research to Integrate Services for the Maintenance of Autonomy method cooperation-item grid as it was used during Programme of Research to Integrate Services for the Maintenance of Autonomy-France to assess the extent of method implementation.

Note: The final rate is calculated on 100 points: 20 for consultation, 20 for case management, 20 for an integrated entry point, 15 for the evaluation tool, 15 for the individualized-services plan and 10 for the information system. The reader can find the entire grid, its development and theoretical justification in [17].

**Table 2.**
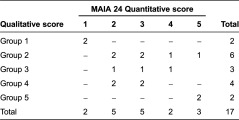
Comparison of groups according to their quantitative MAIA-24 scores versus qualitative data scores. Scores of 1 correspond to the highest level of method implementation, while scores of 5 are the lowest.

## Appendix A: The initial MAIA 24

### Cooperation (four points)

A meeting is held to discuss strategy (1)

If Yes,

Is a meeting frequency established? (1)

If Yes

Decisions are made during the meetings (1)

The hospital is represented at the strategy meeting by a decider (1)

### Single entry point (six points)

No new entry point (for example exclusively dedicated to Alzheimer's disease) is created during the period (1)

An audit is conducted to count the number of responses/entry points (1)

If Yes,

A road map to consolidate requests for assistance is defined among the different entry points (1)

A scripted decision-tree (according to the individual's needs which questions must be asked) is defined and shared among the different entry points (1)

If at least one Yes,

The designation of a single entry point benefits from the pooling of resources (1)

The single entry point is accorded an observatory function for the needs of the population (1)

### Case management (six points)

The presence or absence of case managers is determined (1)

A target population for case management is defined (1)

If Yes for BOTH,

The strategy discussion included case management in its long-term policy (1)

If Yes,

Case load foreseen for case managers <60 (1)

Case-management procedures organize a systematic link with patients’ general practitioners (GPs) (1)

Case-management procedures organize a systematic link with the hospital physician to assure admission and discharge continuity of care (translational care) (1)

### Standardized multidimensional evaluation tool (three points)

An evaluation tool is defined and validated during the strategy discussion (1)

If Yes,

The tool is missing none of the following dimensions: care, functional autonomy, social environment, environment, psychological–cognitive dimension, financial situation (1)

If Yes,

Specific training in the use of the tool is completed (1)

### Individualized services plan (three points)

The services plan can only exist once the evaluation tool has been validated (1)

If Yes,

The tool is missing none of the following dimensions: care, functional autonomy, social environment, environment, psychological–cognitive dimension, financial situation (1)

If Yes,

The needs not covered by the plan are mentioned (1)

### Information system (two points)

No MAIA-dedicated software tool can be developed without the input of the national team (1)

A specifications document, indicating the information that can be exchanged and authorization of access and the network, is defined (1)

### The five groups

If the site had been in the third quartile at the last evaluation, it was classed in category 1: sites that had implemented a major part of the MAIA programme according to the score. If the site had been in the first quartile at the last evaluation, it was assigned to category 5: sites that had poorly implemented the MAIA programme according to the score. If the site was previously rated between those extremes, the progression between the first and last rating was taken into account. If the progression was in the third quartile, it was classed in category 2: sites with a still moderate implementation score but a strong programme-achievement dynamic according to the score. If the progression was in the first quartile, it was designated category 4: sites with a still moderate implementation score and a weak programme-achievement dynamic according to the score. If the progression was between those two extremes, it was classed in category 3: sites with a still moderate implementation score and an intermediate programme-achievement dynamic according to the score.

## Appendix B: The MAIA 25 score

This score was adapted to obtain better differentiation of implementation progress at the different sites. The tool components, individualized services plan and information system were deleted because they did not enable the sites to be distinguished. The progression of these particular components depended more on national politics than local decisions. The single entry point was renamed integrated entry site, which is more acceptable in the French context.

### Cooperation (10 points)

#### (1) Planners’ strategy discussions

A roundtable discussion of strategy took place at least twice during the past year (one point)

If Yes,

The Regional Health Agency and the General Council designated representatives to attend (one point; that the point is attributed only if both agencies have a representative)

If Yes,

Decisions were made during the meetings (one point; note its nature)

Decisions made during those strategy meetings were followed for each structure in the ensuing progress report (one point)

#### (2) Administrators’ tactical discussion

The list of invited participants was validated by the strategy roundtable. It covers public health and social participants, specifies the decision level in the structure and the powers of any representative (one point; the point is attributed only if all the dimensions are satisfied; if any dimension other than ‘representativeness’ is missing, the point is not accorded: no mention of representativeness is interpreted as its absence and does not result in a lost point).

The hospital is represented as tactical cooperation on the medical level (head of the medical pole concerned or his qualified representative) and the management level (director or his qualified representative; one point; the point is attributed only if the two representatives are present; for the sites associated with several hospitals, the presence of a single ‘dual representation’ suffices to obtain the point but is obviously not sufficient with respect to integration).

All operational case-manager teams (social workers, polyvalent and/or geriatric social service, local information centre, local information and coordination centre services, when appropriate) are represented at the roundtable meeting by a representative who is a department head or director (one point; it is attributed only if all the partners are represented).

Decisions made during the tactical discussion are described in the next progress report (one point).

Each participating structure is indeed present and appears in the decision follow-up (one point). (The focus here is on those ‘absent’ from those meetings. Did those with the power to represent them indeed transmit the decisions down the line to the structures? This question should be monitored by the pilot. In the absence of representation, the point is not attributed; indeed, for a decision to be effective, the representation procedures seem necessary, for example, so that all at home nursing services and hospital and temporary lodging services are represented).

Decisions are made based on information from a shared database (one point). (This point could not be attributed during the experimental phase because the partners were not yet sharing information of the integrated entry point -type; at best, these data were collected by the pilot alone).

### Integrated entry site (10 points)

#### (1) Tools

A scripted decision-tree was validated at the strategic and tactical meetings (one point)

The scripted decision-tree is multidimensional (it draws attention to the situation in the fields of health, functional autonomy, and familial and social, economic, administrative, environmental and individual safety needs; one point; it is attributed only if all the dimensions are present; if any dimension is missing and the point is not accorded).

A scripted decision-tree foresees orientations towards a diversified range of services and is not limited to filtering access to care management (one point).

The reference intervention allows orientation towards all territorial resources (one point).

#### (2) Organization

The tactical and strategic roundtable-discussion participants agree to share all the tools of the integrated reception site (one point).

The territoriality of the integrated entry site is defined by written arguments supporting its relevance and validated by the strategy-discussion participants (one point).

Assignment of same-coded tasks and interventions for the national territorial partners is validated at the tactical roundtable discussion (one point).

The tactical and strategic roundtable participants decide on a permanent integrated entry site for information and orientation shared by the partners (one point; this issue concerns rating how partners take one another into account to assure the permanent availability of information: switching telephone numbers to another line, shared resources or personnel and, eventually a single phone number).

Tactical roundtable-discussion decisions rely on the database established via the integrated entry site (one point; the difference with the common database is that the point is attributed if only the pilot uses these data; making these data available to everyone is an additional step).

The documents issued from strategic meetings of case manager and the Regional Health Agency (gerontology diagram, organization plan…) take the MAIA integrated-entry site organization into account (one point).

### Case management (five points)

Case load for each full-time manager is around 40 (one point; the point is attributed for the first 2 years if the case load reaches or exceeds 30; subsequently, it must exceed 36 but be less that 44).

The case-management procedures organize a systematic link with the GP (as attested by the transmission of the ‘case-management procedure’ document (one point).

The case-management procedures organize the place of case managers during hospitalizations and the admission and discharge continuity of care (idem; one point; idem).

The mean time between the first application and the case manager's first visit is <10 days, and the number of missing data for the two dates <10% (one point).

The tactical roundtable-participants’ decisions rely on the data in the case manager's or the database activity report (one point).

## References

[1] Kodner DL (2002). The quest for integrated systems of care for frail older persons. Aging Clinical and Experimental Research.

[2] World Health Organization (2006). The world health report 2006: working together for health.

[3] Strandberg-Larsen M, Krasnik A (2009). Measurement of integrated healthcare delivery: a systematic review of methods and future research directions. International Journal of Integrated Care [serial online].

[4] Kodner DL, Kyriacou (2000). CK Fully integrated care for frail elderly: two American models. International Journal of Integrated Care [serial online].

[5] Hebert R, Durand PJ, Dubuc N, Tourigny A (2003). PRISMA: a new model of integrated service delivery for the frail older people in Canada. International Journal of Integrated Care [serial online].

[6] Leutz WN (1999). Five laws for integrating medical and social services: lessons from the United States and the United Kingdom. Milbank Quarterly.

[7] Hebert R, Raiche M, Dubois MF, Gueye NR, Dubuc N, Tousignant M (2010). Impact of PRISMA, a coordination-type integrated service delivery system for frail older people in Quebec (Canada): a quasi-experimental study. Journal of Gerontology B Psycholocial Sciences Social Sciences.

[8] Somme D, de Stampa M (2011). Ten years of integrated care for the older in France. International Journal of Integrated Care [serial online].

[9] Somme D, Trouve H, Couturier Y, Carrier S, Gagnon D, Lavallart B (2008). Prisma France: programme d'implantation d'une innovation dans un systeme de soins et de services aux personnes en perte d'autonomie. Adaptation d'un modele d'integration base sur la gestion de cas. Revue d'Epidemiologie et de Sante Publique.

[10] Etheridge F, Couturier Y, Trouve H, Saint-Jean O, Somme D (2009). Is the PRISMA-France glass half-full or half-empty? The emergence and management of polarized views regarding an integrative change process. International Journal of Integrated Care [serial online].

[11] Trouve H, Couturier Y, Etheridge F, Saint-Jean O, Somme D (2010). The path dependency theory: analytical framework to study institutional integration. The case of France. International Journal of Integrated Care [serial online].

[12] Somme D, Balard F, Couturier Y, Gagnon D, Saint Jean O, Trouvé H (2011). PRISMA France: projet pilote sur l'intégration et la gestion de cas.

[13] Couturier Y, Trouvé H, Gagnon D, Etheridge F, Carrier S, Somme D (2009). Réceptivité d'un modèle québécois d'intégration des services aux personnes âgées en perte d'autonomie en France pour la revue. Liens social et Politique.

[14] Couturier Y, Bezlile L, Gagnon D (2012). Principes méthodologiques de l'implantation du modèle PRISMA portant sur l'intégration des services pou les personnes âgées en perte d'autonomie. Revue Management et Avenir.

[15] Hebert R, Veil A (2004). Monitoring the degree of implementation of an integrated delivery system. International Journal of Integrated Care [serial online].

[16] Veil A, Hébert R, Hébert R, Tourigny A, Raîche M (2007). Measuring the integration of services between stakeholders in the continuum of services for the elderly in three territories. PRISMA, Volume II: Integration of services for disabled people: research leading to action.

[17] Trouvé H, Veil A, Hébert R, Somme D, Krzysztof S (2010). The PRISMA France study: is there a way to measure implementation of integration in different countries?. Health Management.

[18] Somme D (2014). Integrated care in France: a dream or a reality?. International Journal of Integrated Care [serial online].

[19] Somme D, Trouvé H, Passadori Y, Corvez A, Jeandel C, Bloch M (2014). The French Society of Geriatrics and Gerontology position paper on the concept of integration. International Journal of Integrated Care [serial online].

